# X-Linked Adrenal Hypoplasia Congenita in a Boy due to a Novel Deletion of the Entire* NR0B1 (DAX1)* and* MAGEB1*–*4* Genes

**DOI:** 10.1155/2016/5178953

**Published:** 2016-08-30

**Authors:** Aleksandra Rojek, Maciej R. Krawczynski, Aleksander Jamsheer, Anna Sowinska-Seidler, Barbara Iwaniszewska, Ewa Malunowicz, Marek Niedziela

**Affiliations:** ^1^Poznan University of Medical Sciences, 2nd Chair of Pediatrics, Department of Pediatric Endocrinology and Rheumatology, 27/33 Szpitalna Street, 60-572 Poznan, Poland; ^2^Poznan University of Medical Sciences, Chair and Department of Medical Genetics, Rokietnicka 8 Street, 60-806 Poznan, Poland; ^3^Center for Medical Genetics GENESIS, 4 Grudzieniec Street, Poznan, Poland; ^4^Ludwik Rydygier's Provincial Hospital in Torun, Children's Hospital, Division of Pediatrics, Pediatric Endocrinology and Pediatric Neurology, 42 Konstytucji 3 Maja Street, 87-100 Torun, Poland; ^5^The Children's Memorial Health Institute, Department of Laboratory Diagnostics, 20 Al. Dzieci Polskich, 04-736 Warsaw, Poland; ^6^Karol Jonscher's Clinical Hospital, 27/33 Szpitalna Street, 60-572 Poznan, Poland

## Abstract

X-linked Adrenal Hypoplasia Congenita (AHC) is caused by deletions or point mutations in the* NR0B1 (DAX1) *gene. We present a boy with AHC who came at the age of 25 days in a severe state due to prolonged vomiting and progressive dehydration. Laboratory studies showed prominent hyponatremia and hyperkaliemia but not hypoglycemia. Primary adrenal insufficiency was confirmed with low serum cortisol levels and high plasma ACTH levels. Hydrocortisone therapy combined with saline and glucose infusions was started immediately after blood collection. Two exons of the* NR0B1 (DAX1) *gene were impossible to amplify using the standard PCR method. Array CGH was used to confirm the putative copy-number variation of* NR0B1 (DAX1) *revealing a novel hemizygous deletion encompassing the entire* NR0B1 (DAX1) *gene together with the* MAGEB *genes. This genetic defect was also present in heterozygosity in the patient's mother. We show that* NR0B1 (DAX1) *gene analysis is important for confirmation of AHC diagnosis and highlights the role of genetic counseling in families with AHC patients, particularly those with X chromosome microdeletions, covering more than* NR0B1 (DAX1) *alone. We hope that further clinical follow-up of this patient and his family will shed a new light on the role of* MAGEB *genes.

## 1. Introduction

**Figure 1:**
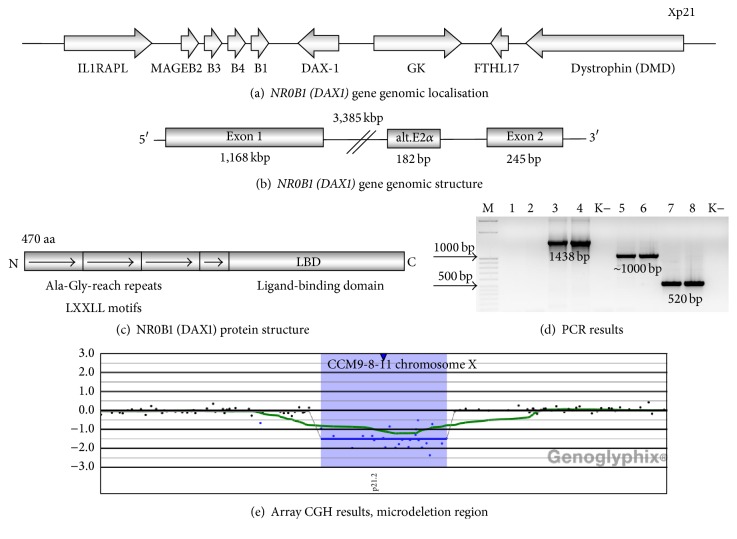
(a) Schematic representation of* NR0B1 (DAX1)* gene localisation in the Xp21 region of the X chromosome between the* IL1RAPL1* and dystrophin (*DMD*) genes.* IL1RAPL1* encodes for a putative transmembrane cytokine receptor related to receptors and receptor accessory proteins for interleukin 1 and interleukin 18 and is expressed in the brain and muscles. The* MAGEB* (melanoma antigen family B) gene cluster encompasses the* MAGEB1*,* B2*,* B3*, and* B4* genes, which are expressed in tumor cells but are silent in normal adult tissues except for the male germ line cells.* NR0B1 (DAX1)*,* GK*: glycerol kinase gene,* FTHL17*: the testis specific ferritin heavy chain gene, and* DMD*: dystrophin gene. (b) Genomic structure of the* NR0B1 (DAX1)* gene composed of two exons (exon 1 and 2). Alternatively spliced exon 2*α* located within the intron is also shown. (c) Functional domain structure of the NR0B1 (DAX1) protein. NR0B1 (DAX1) protein has 470 amino acids and belongs to the nuclear hormone receptor superfamily. NR0B1 (DAX1) displays a novel DNA-binding domain at its amino terminus. This N-terminal repeat region with LXXLL motifs is crucial for protein-protein interactions. The three total repeats and fourth incomplete repeat of a 65- to 67-amino acid sequence, rich in alanine and glycine, are indicated by arrows. The C-terminal part of the NR0B1 (DAX1) protein shows the characteristics of a nuclear hormone receptor ligand-binding domain (LBD) and functions as a transcriptional repression domain. (d) PCR analysis of the* NR0B1 (DAX1)* gene results. M: DNA molecular weight 50 bp marker (Sigma), lanes 1-2: no amplification of exon 1 in the patient, lanes 3-4: amplification of exon 1 in patient's mother, lanes 5-6: amplification of exon 2 in the patient, lanes 7-8: amplification of exon 2 in the patient's mother, and K−: negative control without genomic DNA. Exon 1 was not amplified in the patient, suggesting* NR0B1 (DAX1)* deletion. The higher band that should correspond to exon 2 of* NR0B1 (DAX1)*, based on BLASTn analysis, was similar to chromosome 7. This band was not observed in the patient's mother in whom PCR product of the expected length was amplified (lanes 3-4). (e) Array comparative genomic hybridization (aCGH) results showing a ~300 kb hemizygous deletion on X chromosome encompassing the entire coding sequences of the* NR0B1 (DAX1)* and* MAGEB* genes. Genomic coordinates delineating minimal size of the detected loss according to UCSC genome build HG18 are as follows: chrX: 30017532–30301543. The deletion does not involve neighbouring genes such as* GK*,* DMD*, and* IL1RAPL1*.

X-linked Adrenal Hypoplasia Congenita (AHC, OMIM#300200) is a rare disorder that is characterized by primary adrenocortical failure, occurring due to a lack of permanent adult cortical zone of the adrenal glands. AHC manifests itself mainly in early infancy or in childhood and is caused by deletions or point mutations in the* NR0B1 (DAX1)* (dosage-sensitive sex reversal, adrenal hypoplasia critical region, on chromosome X, gene 1) gene (OMIM*∗*300473) (Figures 1(a)–1(c)) [1].* NR0B1 (DAX1)* deletion may occur in isolation or as part of a contiguous gene deletion syndrome at Xp21 including the dystrophin gene (*DMD*) and the glycerol kinase gene (*GK*). Such mutations result in AHC, Duchenne muscular dystrophy (DMD), and glycerol kinase deficiency (GKD) [2–5]. In some patients loss of the telomeric locus for the interleukin 1 receptor accessory protein-like gene (*IL1RAPL1*) may occur and is associated with developmental delay and intellectual disability [4, 5].

*NR0B1 (DAX1)* is located on the short arm of the X chromosome (Xp21.3-p21.2) and encodes for an orphan nuclear hormone receptor that functions as a transcriptional regulator of other genes [6]. It is expressed in the adrenal glands, gonads, hypothalamus, and pituitary gland and is crucial for their proper development and function. NR0B1 (DAX1) is a transcriptional repressor of other proteins involved in the adrenal steroidogenesis pathway such as steroidogenic factor 1 (SF-1), steroidogenic acute regulatory protein (StAR), P450scc, and 3*β*-hydroxysteroid dehydrogenase [7, 8]. NR0B1 (DAX1) is also involved in sex determination; it antagonizes the action of the testis-determining factor SRY (sex-determining region Y) during gonadal development.* NR0B1 (DAX1)* is expressed in Sertoli cells; thus it is directly involved in the spermatogenesis process. NR0B1 (DAX1) also acts as a shuttling protein between nucleus and cytoplasm. NR0B1 (DAX1) protein binds mRNAs associated with polyribosomes and regulates other gene expressions on the posttranscriptional level [9].

Almost two hundred mutations in the* NR0B1 (DAX1)* gene have been identified to date [2], and the relationship between mutations in the* NR0B1 (DAX1)* gene and AHC is well documented. Frameshift and nonsense mutations may occur within the entire open reading frame of the gene. Missense mutations are not so common and are found mainly in the region encoding for the ligand-binding domain (LBD) at the C-terminus of the NR0B1 (DAX1) protein [3].

Other pathogenic alterations that affect the* NR0B1 (DAX1)* gene comprise copy-number variations (CNVs) of different size. These changes may involve either the entire coding sequence of* NR0B1 (DAX1)* with or without other adjacent genes or are confined to its intragenic fragments (e.g., single exon deletions). To date, such mutations have been described in 22 nonconsanguineous cases, including 17 deletions (HGMD® Professional 2012.3) [2]. AHC is associated with the deletions, which in hemizygous male patients result in a complete loss of the function of NR0B1 (DAX1).

NR0B1 (DAX1) is crucial for the development of the adrenal glands and the hypothalamic-pituitary-gonadal axis; hence its disruption can result in various clinical phenotypes leading to Adrenal Hypoplasia Congenita (AHC). The symptoms often occur early in life, in early infancy or childhood, and include salt loss, hypoglycemia, weak gain of weight, vomiting, prolonged period of postnatal jaundice, skin hyperpigmentation, hyponatremia, hyperkalemia, low level of cortisol, low level of aldosterone, high level of ACTH, and higher plasma renin activity. Patients are usually admitted to hospital in a life-threatening state. AHC can be fatal, unless appropriate steroid replacement therapy is provided to patients immediately [4–8].

Hypogonadotropic hypogonadism (HH) is the most frequently observed puberty disorder that is caused by mutations in the* NR0B1 (DAX1)* gene and is due to impaired gonadotropin synthesis and release. HH is frequently followed by absence or delayed puberty and testosterone replacement therapy is usually introduced to induce sexual maturation in patients with AHC and HH [9–12]. Several rare cases with AHC and normal puberty, peripheral precocious puberty, ACTH-dependent precocious puberty, or central precocious puberty have also been described hitherto [7, 13–16]. Abnormalities in spermatogenesis (oligo-, azoospermia) may also occur in patients with AHC and* NR0B1 (DAX1)* mutations [6, 9, 12, 17, 18]. NR0B1 (DAX1) is also crucial for sex determination.* NR0B1 (DAX1)* gene duplication leads to dosage-sensitive sex reversal (DSS) (46,XY male individuals develop as phenotypic females) and has been found in several patients. Duplications of the X chromosome region containing* NR0B1 (DAX1)* also lead to 46,XY-gonadal dysgenesis or ambiguous genitalia. NR0B1 (DAX1) is known for its “antitestis” function; it acts antagonistically to SRY, WT1, and SF1 and hence inhibits AMH (anti-Müllerian hormone) production. An extra copy of* NR0B1 (DAX1)* can therefore block the development of the male reproductive system [19]. Schematic representation of this gene is shown in Figures 1(a)–1(c) [9, 19–30].

## 2. Aim of the Study

The aim of this study was to investigate the clinical and molecular background of congenital adrenal hypoplasia in a boy, in whom a* NR0B1 (DAX1)* mutation was suspected.

## 3. Case Presentation

The phenotypic boy was born to nonconsanguineous parents in the 41st week of gestation with a birth weight of 3880 g and Apgar score 7/8/10. He was referred and admitted to a Regional Pediatric Hospital in the neonatal period, on the 25th day of life with severe vomiting. He was breast fed and also received extra portions of humanized milk. Immediately after birth he was treated with combined antibiotic therapy since a congenital infection was diagnosed. Physical examination at admission revealed normal skin pigmentation and a skin rash on his trunk. He was apathetic, dehydrated, and cachectic. His weight (4000 g) was almost the same as at the time of delivery. He had normal development of male external genitalia with an intrascrotal location of testes. We found hyponatremia [Na 125.5 mmol/L (ref. 135–145)], hyperkalemia [K 7.98 mmol/L (ref. 3.5–5.0)], and hypochloremia [90.3 mmol/L (ref. 93–112)] but not hypoglycemia [62 mg/dL (ref. 50–99)] in initial biochemical tests. The serum cortisol level of the patient was 444 nmol/L [ref. in the morning >400 nmol/L (150 ng/mL)] however coexisting with an elevated ACTH level [319.0 pg/mL (ref. 10–60)], thereby showing its insufficient release from the adrenal glands in a severe clinical state. The urinary steroid profile (GC/MS) suggested adrenal insufficiency (congenital hypoplasia or bilateral massive adrenal hemorrhage) (Table 1). This test also suggested that there is no adrenal reserve in terms of cortisol secretion. By the clinical and biochemical findings, adrenal insufficiency due to congenital adrenal hypoplasia was diagnosed. The remaining adrenal hormones [17*α*-hydroxyprogesterone 1.8 and 0.8 ng/mL (ref. 0.2–1.6), androstenedione 0.6 ng/mL (ref. 0.1–0.9) and DHEAS <0.40 and 1.27 *μ*mol/L (ref. 1.02–7.16)] as well as testosterone level [1.30 nmol/L (ref. 0.5–14.0)], were all in a wide spectrum of normal range for age and sex. Combined treatment with IV/oral hydrocortisone, oral fludrocortisone, supplementation with saline, and rehydration was used. Abdominal ultrasound imaging excluded pylorostenosis and did not show any organ abnormalities.

## 4. Materials and Methods

### 4.1. Sample Collection, Biochemical Measurements, and Genetic Analysis of the* NR0B1 (DAX1)* Gene

Written informed consent was obtained from the patient and his mother and the study was approved by the local Ethics Committee (number 621/11). Blood samples were collected and frozen at −20°C until analyzed. Genetic analysis of the* NR0B1 (DAX1)* gene was performed in the Molecular Endocrinology Laboratory of the Department of Pediatric Endocrinology and Rheumatology, Poznan University of Medical Sciences, Poland, as well as in the Chair and Department of Medical Genetics University of Medical Sciences in Poznan. Biochemical measurements were performed in the Central Laboratory of K. Jonscher's Clinical Hospital of the University using commercial kits.

### 4.2. Genetic Analysis of the* NR0B1 (DAX1)* Gene

Molecular studies of the* NR0B1 (DAX1)* gene were performed using the standard PCR method. Genomic DNA was isolated from peripheral blood leukocytes using QIAamp® DNA Blood Mini Kit (QIAGEN). Two sets of primers came from Genomed and were used to amplify the coding sequence, 5′UTR and 3′UTR, and flanking intronic sequences of the* NR0B1 (DAX1)* gene. The primer sequences used in this study were described previously [30]. Polymerase chain reactions (PCR) were performed in a 20 *μ*L mixture using HotStarTaq® DNA Polymerase (QIAGEN) under the following parameters: denaturation at 95°C for 15 min, followed by 40–45 cycles of 95°C for 60 s, 53°C (exon 1) or 50°C (exon 2) for 30 s, and elongation in 72°C for 90 s (exon 1) or for 45 s (exon 2). A final amplification at 72°C for 10 min completed the PCR program. PCR products were then separated by electrophoresis on a 1.3% agarose gel in the presence of Ethidium Bromide (Merck) used for visualization. The samples were then purified from the gel using QIAquick® Gel Extraction Kit (QIAGEN) and directly sequenced with the same primer pairs used in the PCR. Sequencing reactions were performed with a BigDye Terminator v3.1 cycle sequencing kit (Applied Biosystems) on an ABI Prism 3130XL Genetic Analyzer (Applied Biosystems). Finally, the sequences were analyzed using Vector NTI9.0 Software (Invitrogen) and compared to the NCBI Reference Sequence NG_009814.1 (22.07.2010). BLASTn was used in analysis of the sequence of PCR product obtained in the patient that should correspond to exon 2 of the* NR0B1 (DAX1)* gene.

### 4.3. Array Comparative Genomic Hybridization (aCGH)

Array comparative genomic hybridization (aCGH) was performed using a CGX format NimbleGen 12-plex microarray with a resolution of 135 k (134829 oligonucleotide probes) per haploid genome. CGX chips are designed to target gene-rich chromosomal regions at the highest density. DNA of the index case as well as commercially available reference DNA (Promega®) were differentially labeled with fluorescent dyes (Cy3 and Cy5, resp.) and cohybridized onto the array slide according to the manufacturer's protocols (NimbleGen). The array was subsequently scanned with the use of a Roche NimbleGen platform (MS 200 Microarray Scanner). Data generated on scanning was extracted by NimbleScan Software (Roche NimbleGen) and then analyzed for clinical relevance by means of Genoglyphix® Software (Signature Genomics).

## 5. Results

### 5.1. The Result of Genetic Analysis of the* NR0B1 (DAX1)* Gene

PCR amplification of the* NR0B1 (DAX1)* gene showed no amplification of exon 1 in the AHC patient (Figure 1(d), lanes 1 and 2). Sequencing of the PCR product obtained in the presence of primers specific for exon 2 of the* NR0B1 (DAX1)* gene showed a 92% identity of the sequence with human chromosome 7. This product was obtained due to the deletion of the* NR0B1 (DAX1)* gene and hybridization of the primers for exon 2 to their complementary sequences located in chromosome 7. Using standard PCR method we were not able to define if the patient's mother is a carrier of the mutation or not (Figure 1(d), lanes 3 and 4).

### 5.2. Array Comparative Genomic Hybridization (aCGH) Results

**Figure 2:**
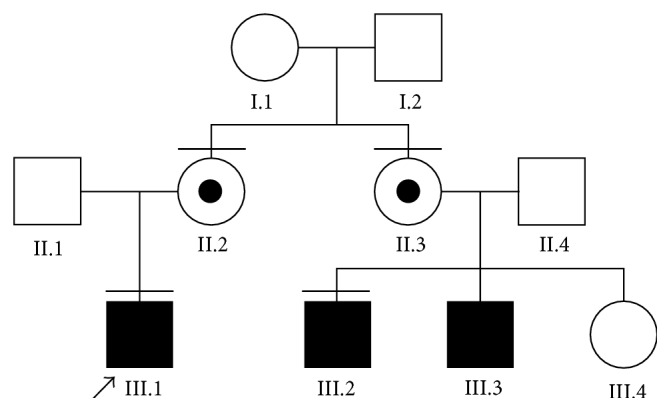
Pedigree of the family. Horizontal bars above the symbols represent availability for genetic testing.

Array comparative genomic hybridization revealed a loss of 24 consecutive oligonucleotide probes, thus showing hemizygous interstitial deletion on chromosome Xp21.2. Minimal size of the detected CNV encompassed 284.01 (genomic coordinates 30017532–30301543 according to HG18), whereas estimated maximum size was 329.80 kb (Figure 1(e)). The deletion involved the five following genes:* MAGEB2*,* MAGEB3*,* MAGEB4*,* MAGEB1*, and* NR0B1 (DAX1)*. Neighbouring genes such as* GK* and* IL1RAPL1* were unaffected by the CNV. Additional analysis showed that the patient's mother was a carrier of the deletion. Furthermore, a younger maternal cousin affected by AHC and his mother (a healthy sister of the mother of our proband) were also demonstrated to carry identical deletion (Figure 2).

## 6. Discussion

In this study we present a boy with AHC, with normal testes and normal male external genitalia development in whom a novel type of deletion in the* NR0B1 (DAX1)* gene comprising the entire coding sequence of the* NR0B1 (DAX1)* was detected. This deletion resulted in a complete loss of function of the NR0B1 (DAX1) protein. The* NR0B1 (DAX1)* gene defect coexisted with the deletion of other neighbouring genes (*MAGE* family genes) and resulted from a submicroscopic microdeletion of the short arm of chromosome X. The role of the* MAGE* genes (*MAGEB1*,* MAGEB2*,* MAGEB3*, and* MAGEB4*) is still unknown; however it is known that they are expressed in embryonic tissues, in some tumors, and after birth only in the testes [31]. The microdeletion of the short arm of chromosome X found in our patient does not include the* DMD* gene; therefore the features of muscular dystrophy should not appear in the future.

X-linked AHC is caused predominantly by point mutations in the* NR0B1 (DAX1)* gene; however it can also result from its partial or complete deletion. Genomic rearrangements may account for either classical AHC or a phenotype with additional features, depending on the CNV size and number of genes that are affected. In the patient presented here, AHC was caused by a pathogenic deletion encompassing the* NR0B1 (DAX1)* and four* MAGE* family genes. Therefore, we suggest that quantitative screening, preferably array CGH, should be included in the molecular testing of AHC cases, especially if the sequencing fails to detect causative alterations. Unlike other targeted methods, such as qPCR or MLPA, array CGH allows for both confirmation of* NR0B1 (DAX1)* deletion and precise delineation of its size, which is extremely important for proper counseling and clinical management of affected individuals.

Most patients with X-linked AHC present adrenal insufficiency early in life and have combined glucocorticoid and mineralocorticoid deficiency [32]. AHC is also characterized by puberty failure and hypogonadotropic hypogonadism as well as growth hormone deficiency later in life [33]. Individuals with milder forms of AHC have also been described, but the incidence is not common. In such patients adrenal insufficiency is incomplete and their sexual maturation is delayed or arrested due to mild gonadotropin deficiency [18, 23, 33, 34]. These milder clinical phenotypes result from incomplete* NR0B1 (DAX1)* loss-of-function mutations, mostly including missense variants. For instance, two such mutations in the NR0B1 (DAX1) carboxy terminal region were described (I439S, Y380D) with partial repression activity of SF1-mediated transcription [18, 35]. In turn, a Q37X nonsense mutation in NR0B1 (DAX1) led to an alternative translation of the NR0B1 (DAX1) protein generated from an alternate in-frame translation initiation site (codon 83 for methionine) [23]. Translation of this truncated isoform of the NR0B1 (DAX1) protein, possibly exerting some residual activity, might prevent the patient from the classical AHC phenotype and delay the onset of adrenal insufficiency until early adulthood [23].

## 7. Conclusions

The clinical signs and symptoms of AHC are similar to that observed in patients with the salt wasting form of congenital adrenal hyperplasia caused by 21-hydroxylase deficiency; thus it is very important to distinguish between these two disorders, because they have a different clinical course and implications for genetic counseling. Here we presented a boy with a novel deletion of the entire* NR0B1 (DAX1)* gene, associated with congenital adrenal insufficiency and normal development of male external genitalia.

This report thus highlights the value of* NR0B1 (DAX1)* genetic analysis in confirming an AHC diagnosis and emphasizes the critical role of genetic counseling in families with AHC patients, particularly in those with a significant deletion of X chromosome material including the* NR0B1 (DAX1)* gene.

## Figures and Tables

**Table 1:** The patient's urinary steroid profile by gas chromatography/mass spectrometry (GC/MS) (values 0.0 < the sensitivity of the method).

Compound name	Result (*μ*g/d)	Normal range
AN	2.6	1–10
ET	0.6	1–5
11-OAN/ET	1.1	5–20
11-OAN	1.1	5–20
11-OHET	0.0	
DHA^1^	0.0	1–10
5-AND^1^	0.0	
16a-OHDHA^1^	0.7	135–500
An-3-ol^1^	0.0	40–600
5-PT^′1^	0.0	2–20
16-OHPN^′1^	0.0	110–495
17-OHPN (5b)	0.6	4–19
17-OHPN (5a)	0.0	
PT	0.5	5–21
PTN	0.0	0–5
PD	0.4	2–20
E1	0.0	
E2	0.0	
E3	0.0	
THS	**6.1↑**	1–3
THDOC	0.0	
THA	0.0	5–30
THB	0.0	
Allo-THB	0.0	
18-OHTHA	0.0	
THAldo^2^	**1.8↓**	4.3–12.3
18-Oxo-THF	0.0	
18-OHF	0.0	
THE^3^	73.3	38–408
THF^3^	0.0	
Allo-THF^3^	0.0	
a-CTN^3^	1.1	20–100
b-CTN^3^	15.0	20–100
b-CT^3^	0.0	5–20
a-CT^3^	0.0	5–20
E^3^	10.1	5–20
F^3^	**0.0↓**	3–20
6b-OHF^3^	0.0	
20a-DHF^3^	0.0	

^1^Foetal adrenal steroid metabolites.

^2^Aldosterone metabolite.

^3^Cortisol metabolites.
